# Thermally triggered release of the bacteriophage endolysin CHAP_K_ and the bacteriocin lysostaphin for the control of methicillin resistant *Staphylococcus aureus* (MRSA)

**DOI:** 10.1016/j.jconrel.2016.11.030

**Published:** 2017-01-10

**Authors:** Hollie Hathaway, Jude Ajuebor, Liam Stephens, Aidan Coffey, Ursula Potter, J. Mark Sutton, A. Toby A. Jenkins

**Affiliations:** a Department of Chemistry, University of Bath, BA2 7AY, UK; b Department of Biological Sciences, Cork Institute of Technology, T12 P928, Ireland; c Microscopy and Analysis Suite, University of Bath, BA2 7AY, UK; d Technology Development Group, Public Health England, Porton Down, SP4 0JG, UK

**Keywords:** PNIPAM, Bacteriophage endolysin, Bacteriocin, Thermal release

## Abstract

*Staphylococcus aureus* infections of the skin and soft tissue pose a major concern to public health, largely owing to the steadily increasing prevalence of drug resistant isolates. As an alternative mode of treatment both bacteriophage endolysins and bacteriocins have been shown to possess antimicrobial efficacy against multiple species of bacteria including otherwise drug resistant strains. Despite this, the administration and exposure of such antimicrobials should be restricted until required in order to discourage the continued evolution of bacterial resistance, whilst maintaining the activity and stability of such proteinaceous structures. Utilising the increase in skin temperature during infection, the truncated bacteriophage endolysin CHAP_K_ and the staphylococcal bacteriocin lysostaphin have been co-administered in a thermally triggered manner from Poly(*N*-isopropylacrylamide) (PNIPAM) nanoparticles. The thermoresponsive nature of the PNIPAM polymer has been employed in order to achieve the controlled expulsion of a synergistic enzybiotic cocktail consisting of CHAP_K_ and lysostaphin. The point at which this occurs is modifiable, in this case corresponding to the threshold temperature associated with an infected wound. Consequently, bacterial lysis was observed at 37 °C, whilst growth was maintained at the uninfected skin temperature of 32 °C.

## Graphical abstract

**Figure:**
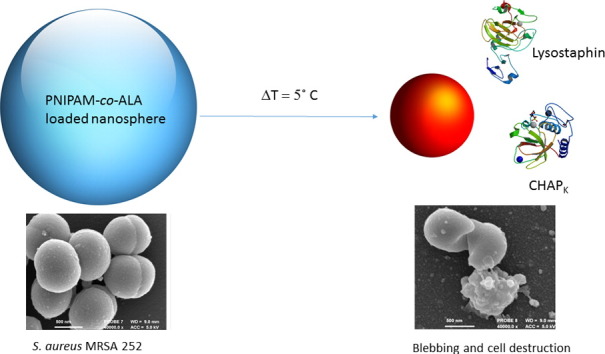

## 1 Introduction

*Staphylococcus aureus* (*S. aureus*) is a frequent inhabitant of the human skin flora colonizing up to 30% of individuals at any given time, primarily through nasal carriage [1]. Requiring a suitable portal of entry into the body, the skin normally provides such a barrier to progressive infection. However a breach in the skin, often as a result of a scratch, cut or burn provides a suitable infection point for the opportunistic bacteria. *S. aureus* is the leading cause of skin and soft tissue infections (SSTI) across all continents, thus resulting in both delayed wound healing and further systemic infections, such as sepsis, osteomyelitis and endocarditis [2]. With the discovery of staphylococcal drug resistance and the subsequent global epidemic that methicillin resistant *S. aureus* (MRSA) has become, the need to source alternative treatment has become paramount. Hospital acquired MRSA (HA-MRSA) has a mortality rate twice that of its methicillin susceptible counterpart, and is more than twice as expensive to treat [3]. Furthermore, the isolation of these ‘super-bugs’ is not confined to the hospital setting. Indeed, community acquired MRSA (CA-MRSA) is proving an equal challenge to clinicians worldwide [4].

Bacteriophage (phage), (the naturally occurring parasitic viruses of bacteria, able to infect and destroy bacterial cells) were first utilised as a treatment to infection in the 1930s within the former Soviet Union. Despite the continued development of phage products throughout the Cold War, bacteriophage therapy was largely disregarded in the West from under the relative comfort of the antibiotic blanket. However, the alarming rise in multi-drug resistance (MDR) in recent times has regenerated interest in phage therapy [5]. One of the main disadvantages associated with the use of whole phage to treat infection is the viral nature of the phage itself. Containing a vast amount of genetic material, temperate phage have been known to increase the virulence of certain species of bacteria through transduction, an example of which includes the bacterial acquisition of the gene encoding the Panton Valentine Leucocidin toxin, causing ‘scalded skin syndrome’ [6]. Whilst this is selected against when sourcing phage for treatment, the regulation and control of suitable virulent phage for clinical use is often timely and uncertain.

Bacteriophage-encoded endolysins (peptidoglycan hydrolases synthesised by phage infected bacterial cells) are utilised in the end stages of phage infection. Lysins are capable of destroying the bacterial cell wall through digestion of the peptidoglycan polymer, resulting in cell death through osmolysis [7]. The isolation of these hydrolases has the potential to overcome many issues surrounding the use of whole phage. As hydrolytic enzymes, they retain specificity without affecting commensal flora, are capable of rapid bacterial lysis, are unlikely to encounter resistance owed to the essential bacterial binding sites and they do not contain transducible genetic information [8]. The specific mechanism of action of these endolysins is discussed elsewhere [9]. Endolysins demonstrating activity towards both Gram-positive and Gram-negative bacteria have been isolated and characterised, including lysins active against *Acinetobacter baumanni*, *Bacillus anthracis*, *Streptococcus pyogenes* and in some cases active against both Gram-positive and –negative bacteria simultaneously [10], [11], [12]. The phage endolysin designated LysK isolated from the staphylococcal bacteriophage K has been shown to have potent antimicrobial activity against a range of staphylococci including MRSA [13]. LysK has been truncated to its single catalytic domain, a cysteine, histidine-dependent aminohydrolase/peptidase (CHAP_K_). This single domain, 18.6 kDa antimicrobial enzyme has been fully characterised and has demonstrated retention of lytic activity in vitro, in vivo and against staphylococcal biofilms [14], [15], [16].

Another class of potential alternative antimicrobials are bacteriocins. Lysostaphin, a 26.8 kDa metalloendopeptidase is produced naturally by *Staphylococcus simulans* [17]. Consisting of a single lytic domain (glycyl-glycine M23 endopeptidase), lysostaphin demonstrates potent antistaphylococcal activity through cleavage of the pentaglycine cross-bridges within the peptidoglycan of the bacterial cell wall. Active against a multitude of antibiotic susceptible, intermediate and resistant strains of bacteria, lysostaphin exhibits synergistic behaviour with multitude of antibiotics, phage lysins and antimicrobial peptides [18], [19], [20]. Successful application of lysostaphin has been demonstrated in cases of ocular infection, osteitis and endocarditis [21], [22], [23].

Despite the discovery and development of new potential antimicrobial candidates, the mode of delivery of any such pharmacologically active substance remains equally as important as the discovery itself. Ensuring activity, stability and dosage conditions are correct (especially when considering biological material), is crucial to successful administration in order to maximise the therapeutic benefit and to reduce any potential side effects. The triggered release of a therapeutic agent (small molecule, protein or virus) may rely on a variety of external stimuli in order to release the active cargo, including pH, temperature, ultrasound, magnetism or biomarker signals [24], [25]. Utilising the difference between the healthy and the diseased state may provide certain conditions whereby treatment can be administered in a controlled fashion. Consequently, high local concentrations are achieved only in the specific location required. When considering treatment of bacterial infection, preventing the administration of unnecessary or sub-lethal concentrations of an antibiotic or antimicrobial agent is crucial in preventing the continued development of antibiotic resistance [26].

Poly(*N*-isopropylacrylamide) (PNIPAM) has been widely investigated as a triggered drug release vehicle [27]. As a thermoresponsive polymer, PNIPAM undergoes a reversible, entropically driven phase transition at a lower critical solution temperature (LCST), resulting in the expulsion of water and a subsequent change in polymer volume. The LCST of PNIPAM and its associated structures (nanoparticles, micelles, nanogels etc.) can be manipulated through control of polymer concentration, copolymers and surfactants. Through adjusting the LCST to that of a clinically relevant temperature, PNIPAM has been extensively investigated in a wide range of biomedical applications including cancer therapy, wound healing, bioscaffolding and cell cultivation [28], [29], [30], [31].

In previous studies, PNIPAM nanoparticles were formulated with allylamine for the controlled release of Bacteriophage K, which demonstrated potent antistaphyloccal activity through the thermally controlled collapse of the nanoparticles [32]. However in this study, phage virions were replaced with a synergisitic enzybiotic cocktail. Allylamine was used in order to adjust the LCST to a biologically relevant temperature of 34 °C, which is indicative of an infected wound. A bacterial infection of a wound as been shown to present as an elevation in skin temperature of around 3.6 °C (demonstrated in infected leg ulcers), in comparison to the surface temperature of approximately 32 °C seen in healthy skin [33]. PNIPAM nanoparticles were anchored to non-woven polypropylene to simulate a wound dressing using plasma deposited maleic anhydride and free amine groups from allylamine. Plasma based deposition strategies for surface activation have been well documented elsewhere [34], [35], [36]. As a representative bacterial isolate, MRSA252 was chosen, which is a HA-MRSA bacterium belonging to the clinically relevant epidemic MRSA-16 clone (EMRSA-16), and considered endemic in the majority of UK hospitals [37].

## 2 Materials and methods

### 2.1 Materials

*N*-isopropylacrylamide, allylamine, ethylene-glycol diacrylate, sodium persulfate, sodium dodecylsulfate (SDS), phosphate buffered saline (PBS) tablets (pH 7.4), vancomycin hydrochloride from *Streptomyces orientalis* and maleic anhydride were all purchased from Sigma-Aldrich (Poole, Dorset, UK).

Lysostaphin from *Staphylococcus simulans*, Tryptic Soy Broth (TSB) and Tryptic Soy Agar (TSA) were purchased from Sigma-Aldrich (Poole, Dorset, UK). Coomassie (Bradford) Protein Assay Kit was purchased from Pierce Scientific. MRSA 252 was sourced from a bacterial strain collection belonging to the Biophysical Chemistry Research Group housed at the University of Bath.

### 2.2 Material preparation

#### 2.2.1 PNIPAM nanoparticle synthesis, plasma deposition and surface grafting

Particles were synthesised via precipitation polymerisation and anchored onto of non-woven polypropylene (2 × 2 cm) squares via plasma deposited maleic anhydride as previously described [32], the only modification being an increase in the surface grafting duration from 1 to 24 h. Following surface attachment, samples were washed in DI water, air dried and kept under aseptic conditions prior to enzyme addition.

#### 2.2.2 Electron microscopy

Samples were prepared as above, freeze dried, sputter coated with gold and imaged via a Scanning Electron Microscope (SEM) (JEOL JSM6480LV operated at 10 KV).

### 2.3 Microbiology

#### 2.3.1 Bacteria and growth conditions

MRSA 252 was taken from freezer stock (stored as a 15% (*v*/v) glycerol at − 80 °C), streaked across a TSA culture plate with a loop spreader and incubated at 37 °C overnight in order to obtain single colonies. Bacterial cultures were prepared by inoculating 10 ml TSB with a single colony and incubating at 37 °C with agitation overnight.

#### 2.3.2 CHAP_K_ production

CHAP_K_ production was performed as previously described [16]. Briefly, the truncated lysin CHAP_K_ was previously cloned and expressed using a pQE60 vector (Qiagen) in *Escherichia coli* (*E. coli*) XL1-Blue [38]. This recombinant *E. coli* was grown at 37 °C with shaking. Protein expression was achieved by inducing the cells with Isopropyl β-D-1-thiogalactopyranoside (IPTG). After which, the cells were lysed and active CHAP_K_ was purified to > 90% homogeneity by cation exchange chromatography and quantified via the Bradford assay [39].

#### 2.3.3 Minimum inhibitory concentration (MIC)

MICs for both CHAP_K_ and lysostaphin were determined by the classical microdilution broth method, conducted according to the Clinical and Laboratory Standards Institute (CLSI) guidelines [40], [41]. Briefly, MRSA 252 cells at 7 × 10^5^ colony forming units per millilitre (CFU/ml) were added to wells containing varying concentrations of CHAP_K_ (64–1 μg/ml) and lysostaphin (0.25–0.004 μg/ml) in a microtitre plate. The plate was then incubated for 18 h with shaking in a micro-plate reader (SPECTROstar Omega, BMG LABTECH) and bacterial growth monitored as a function of optical density (OD) at 600 nm. Experiments were conducted in triplicate both at 37 °C and 32 °C. Deionised (DI) water was used to make enzyme stock solutions and for control experiments.

#### 2.3.4 Turbidity reduction assays

The in vitro activity of both CHAP_K_ and lysostaphin was assessed according to the rate of bacterial cell lysis. MRSA 252 cells at 7 × 10^5^ CFU/ml were grown to an OD of 0.5, centrifuged at 4000 rpm for 20 min at 4 °C, washed twice and resuspended in PBS to reattain an OD of 0.5. A range of CHAP_K_ and lysostaphin solutions were prepared in deionised water with concentrations relative to their MIC (0–16%) in order to assess enzyme activity in the presence of excess substrate: 100 μl of enzyme solution was added to 100 μl of bacterial solution in a microtitre plate in triplicate, the plate was shaken for 5 s to ensure sufficient mixing and the reduction in OD at 600 nm over 5 min was monitored using a micro-plate reader at either 37 °C and 32 °C. Control experiments were undertaken using deionised water in place of either CHAP_K_ or lysostaphin.

#### 2.3.5 Synergy

Synergy was assessed according to the checkerboard assay [42]. Briefly, 100 μl of 7 × 10^5^ CFU/ml MRSA 252 was added to each well of an 8 × 8 section of a 96 well microtitre plate containing varying ratios of CHAP_K_: lysostaphin, ranging from 64 to 1 μg/ml CHAP_K_ and 0.25–0.004 μg/ml lysostaphin. The plate was incubated at 37 °C for 24 h without shaking and bacterial growth assessed visually. Synergy was confirmed by observation of wells with no visible bacterial growth at enzyme concentrations lower than the MICs of the individual antimicrobials.

#### 2.3.6 Electron microscopy

Bacterial samples were grown on Melinex® film overnight in 5 ml TSB at 37 °C with minimal agitation (70 rpm). The volume of growth media was adjusted to 2 ml and exposed to either 64 μg/ml CHAP_K_, 0.125 μg/ml lysostaphin, a combination of 8 μg/ml + 0.031 μg/ml lysostaphin or DI water (control) for 10 min at 37 °C. Samples were then fixed with 2.5% glutaraldehyde in 0.1 M sodium cacodylate buffer, postfixed in aqueous 1% osmium tetroxide, dehydrated in an acetone series (50–100%) and chemically dried in hexamethyldisilazane (HMDS). Samples were sputter coated with a thin layer of chromium and imaged using a Field Emission Scanning Electron Microscope (FESEM) (JEOL JSM6301F operating at 5 KV).

### 2.4 Controlled release experiments

#### 2.4.1 Addition of CHAP_K_ and lysostaphin to anchored nanoparticles

A cocktail consisting of 80 μg/ml CHAP_K_ and 0.31 μg/ml lysostaphin (10 times the concentration shown to generate synergistic inhibition) was incorporated into the anchored gel matrix via soaking the dried gel modified polypropylene fabric in 500 μl of enzyme solution for 2 h at room temperature (20 °C). The fabric was washed in DI water and air dried. Control experiments were undertaken using DI water in place of enzyme solution. In order to estimate protein loading of the nanoparticles, the Bradford assay was used to quantify total protein concentration both in the soaking solution (as confirmation of synergy concentration) and in the solution in which the fabric swatches were washed post soaking. All experiments were performed in triplicate and corrected against control swatches (soaked in DI water) in order to eliminate any non-protein associated solution absorbance. An average blank reading was used to baseline correct all absorbance readings, using DI water only. The residual protein concentration post washing was subtracted from the initial soaking concentration in order to estimate loading efficiency (LE) according to the following equation:

$$\mathrm{LE}\;\left(\%\right)=\frac{\left(\text{total protein added}-\text{protein in washings}\right)}{\text{total protein added}}\times 100$$

#### 2.4.2 Thermal release of CHAP_K_/lysostaphin cocktail from grafted pnipam nanoparticles

Polypropylene-nanoparticle swatches incorporating the enzybiotics were soaked individually in 250 μl of MRSA 252 of fixed concentration (1.2 × 10^8^ CFU/ml in PBS) at either 32 °C or 37 °C for 30 min. Each square of fabric was then removed from solution and placed into 24.75 ml DI water (diluting the original solution volume 100 fold) alongside any residual liquid that had not been soaked up by the fabric. The 25 ml culture tube containing the fabric swatch was vortexed to remove any bacteria attached to the fabric and the solution from each tube was diluted and plated (in triplicate) on TSA plates and incubated at 37 °C overnight. Plates were assessed the following day for growth and any colonies counted. Control experiments were undertaken using the swatches without enzyme and each individual experiment was repeated 6 times.

## 3 Results and discussion

### 3.1 Material analysis

#### 3.1.1 PNIPAM nanoparticle characterisation and surface grafting

The PNIPAM nanoparticles have been characterised by dynamic light scattering (DLS) and according to zeta potential analysis as reported in a previous study [32]. The LCST of the nanoparticles was shown to be transitional but clearly definitive at 34 °C, manifesting as a change in the hydrodynamic radius from 400 nm ± 50 nm in the expanded state (< 33 °C), to 170 nm ± 30 nm in the collapsed state (> 35 °C). Therefore the collapse of the nanoparticles and subsequent release of the enzybiotic cargo is targeted to occur only at a higher temperature associated with bacterial wound infection (around 36 °C), whilst remaining intact and preventing the release of the antimicrobial payload at temperatures indicative of an uninfected wound (around 32 °C). Successful deposition of maleic anhydride onto the non-woven polypropylene as anchor points for the nanoparticles was confirmed by Fourier Transform Infrared Spectroscopy (FT-IR) (S1-Supporting Information) as previously described.

#### 3.1.2 Electron microscopy

**Fig. 1:**
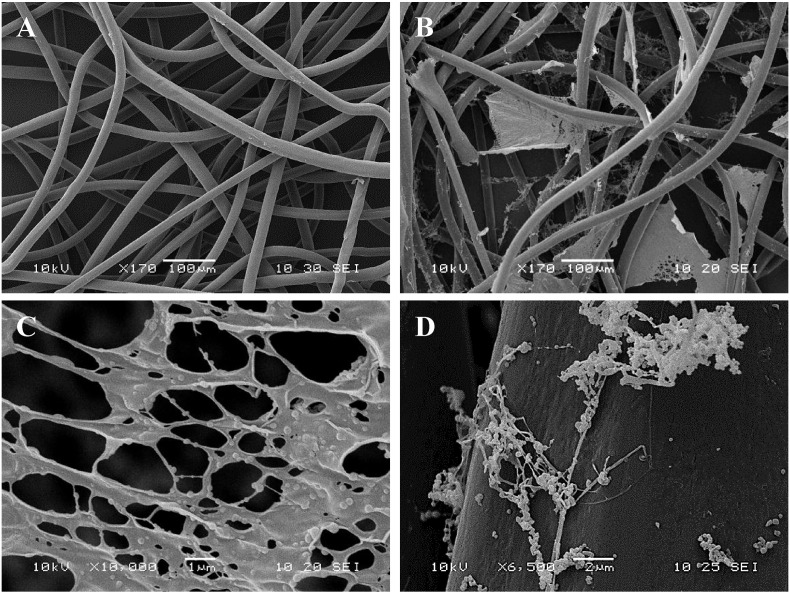
SEM images of non-woven polypropylene fabric (A) Untreated (B) Following PNIPAM nanoparticle attachment (C) Polymeric matrix seen dispersed within the fibre network (D) Nanoparticles attached to the surface of a polypropylene fibre.

SEM images indicated the incorporation of the PNIPAM nanoparticle solution to non-woven polypropylene following surface activation with plasma deposited maleic anhydride and wash steps, as shown in Fig. 1A and B. The large polymer webs (Fig. 1B) seen throughout the fibre matrix, appear to consist of individual nanoparticles held together by the polymeric sheets (Fig. 1C). The nanoparticles are of varying sizes encompassing both the swollen and the collapsed state, possibly as a result of the freeze drying during the sample preparation. Fig. 1D shows the surface attachment of individual nanospheres.

### 3.2 Microbiological analysis

#### 3.2.1 Minimum inhibitory concentration (MIC)

The MIC was determined at both 32 °C and 37 °C as 64 μg/ml and 0.125 μg/ml, for CHAP_K_ and lysostaphin, respectively, illustrating no change in the ability of either enzyme to prevent bacterial growth within this temperature range.

#### 3.2.2 Turbidity reduction and rate of cell lysis

**Fig. 2:**
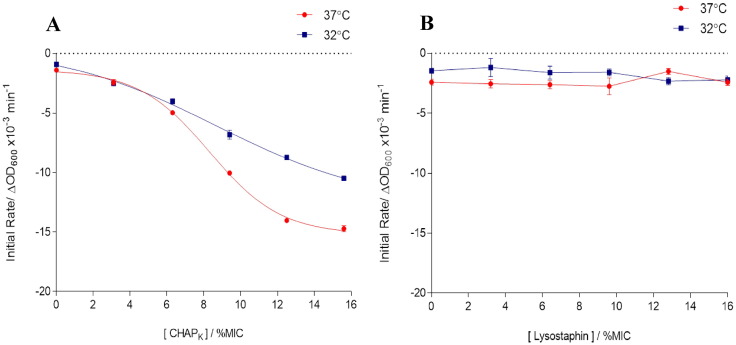
Comparison of the initial rate of bacterial cell lysis by: (A) CHAP_K_ (B) lysostaphin.

The rate at which both CHAP_K_ and lysostaphin catalyse cell lysis was determined by assessing the reduction in turbidity (OD) of a bacterial suspension over the course of 60 s, defined as the initial rate of reaction (S2-Supporting Information). This was investigated at 32 °C (below the LCST of the nanospheres) and at 37 °C (above the LCST), and at low concentrations of enzyme in order to prevent substrate concentration from becoming a limiting factor. Initial rates were calculated by means of tangents and plotted as a function of enzyme concentration relative to the MIC (Fig. 2A and B).

From these results it can be seen that CHAP_K_ is capable of eliciting a rapid reduction in turbidity (associated with bacterial cell lysis) which follows a standard dose response at both temperatures, whilst slightly more linear at 32 °C. There is a small difference in the initial rate of reaction at the two temperatures, more noticeably at higher concentrations of CHAP_K_ but not to such an extent that indicates enzyme activity is substantially hindered at the lower temperature. However it appears that lysostaphin is unable to initiate a substantial degree of cell lysis (even at the highest concentrations) in such a short timeframe despite having a very low overall MIC, with values being similar to that of the control and demonstrating little temperature associated effect. This is in keeping with previous studies whereby CHAP_K_ exhibited a greater reduction in turbidity over a 5 min period when compared to lysostaphin of the same absolute concentration (independent of MIC) [14].

#### 3.2.3 Assessment of synergistic behaviour

**Fig. 3:**
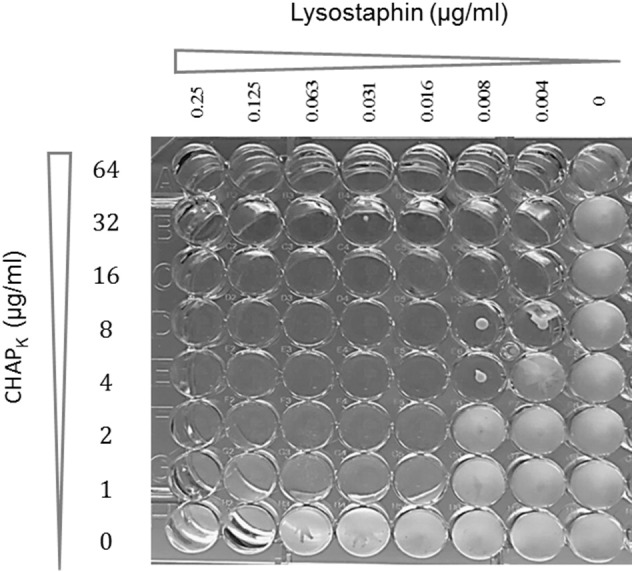
Synergy analysis of CHAP_K_ (MIC = 64 μg/ml) (ordinate) and lysostaphin (MIC = 0.125 μg/ml) (abscissa).

Synergy was assessed both visually (non-growth of bacteria in well plate) and via calculation of fractional inhibitory concentration (FIC), taking the value of the MIC of the enzymes in combination, divided by the MIC of the individual enzyme. To achieve strong synergy the sum of the two FICs (ΣFIC = FIC_A_ + FIC_B_) must be < 0.5 [43]. As shown in Fig. 3, synergy is indicated across the microtitre plate, at a range of different concentrations and confirmed in a total of 11 wells with FICs ranging from 0.144 (well G5) to 0.378 (well C5). A combination of 8 μg/ml CHAP_K_ and 0.031 μg/ml lysostaphin was chosen as a concentration which demonstrates a strong synergistic combination (corresponding to a 3 fold reduction in the MIC of lysostaphin and a 4 fold reduction in the MIC of CHAP_K_), in order to utilise the ability of lysostaphin to inhibit bacterial growth at low concentration, together with an increased rate of cell lysis exhibited by CHAP_K_.

#### 3.2.4 Electron microscopy

**Fig. 4:**
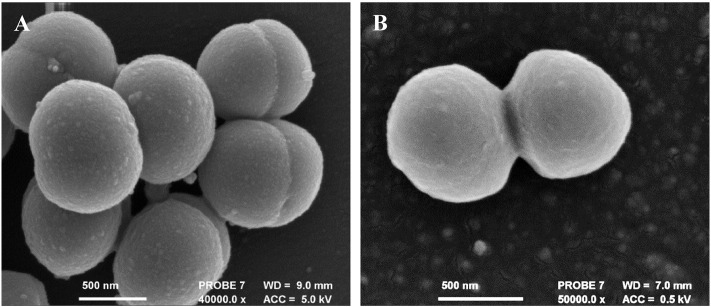
SEM images of untreated and antibiotic treated *S. aureus* MRSA 252 cells (A) cells exposed to DI water (control) (B) cells exposed to 2 × MIC (12.5 μg/ml) vancomycin. 10 min incubation time. Cell division observed in both cases.

Untreated MRSA cells exhibited uniformity in the presentation of their cell morphology as shown in Fig. 4A, active cell division was also observed. Cells treated with vancomycin at twice the MIC (12.5 μg/ml) for 10 min as shown in Fig. 4B, did not present any change in their surface morphology or exhibit any visible membrane damage.

**Fig. 5:**
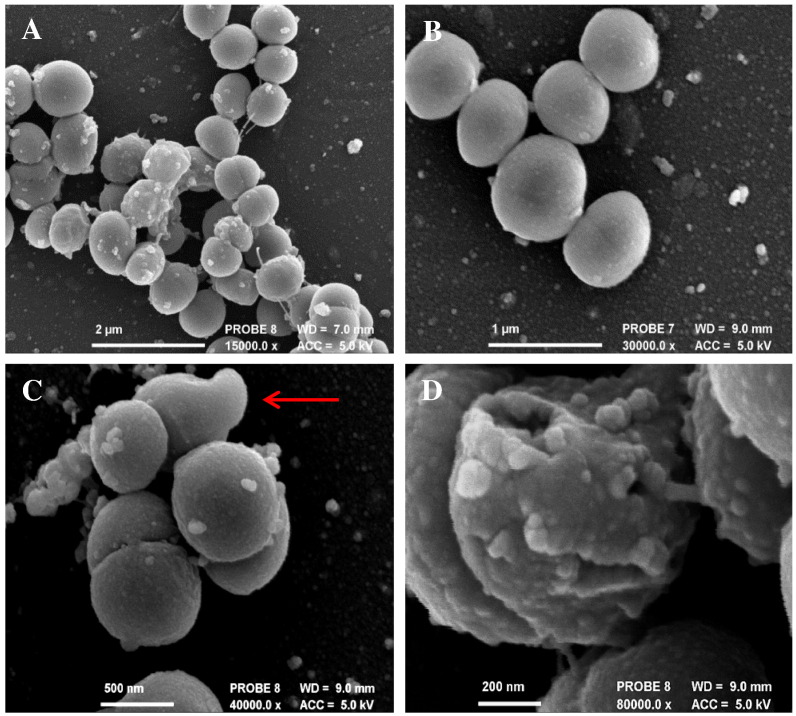
SEM images of *S. aureus* MRSA 252 cells treated with antimicrobial enzymes (A) At MIC: 64 μg/ml CHAP_K_ (B) At MIC 0.125 μg/ml lysostaphin (C + D) Sub-individual MIC 8 μg/ml CHAP_K_ + 0.031 μg/ml lysostaphin. 10 min incubation time.

Cells treated with CHAP_K_ (at the MIC) (Fig. 5A) show some surface damage and slightly more extracellular debris when compared to the control (Fig. 4A); however the majority of the cells appear unaffected. When evaluating cells exposed to lysostaphin at the MIC (Fig. 5B), they are comparable in appearance to those in the control group. This is in keeping with the established rate of lysis observed in solution, with CHAP_K_ being much faster in eliciting a response. The synergistic effect of the cocktail of the two enzymes can be clearly observed in Fig. 5C: blebbing (protrusion of the plasma membrane) was seen (red arrow) and a high degree of debris. Fig. 5D also shows clear evidence of cell lysis by the enzybiotic cocktail.

### 3.3 Controlled release

Fabric swatches with surface anchored PNIPAM nanoparticle gel were soaked in an enzyme solution consisting of 80 μg/ml CHAP_K_ and 0.31 μg/ml lysostaphin (10 times the synergy MIC), this was confirmed using the Bradford assay. The unencapsulated, residual protein concentration in solution post soaking and washing was calculated (Table 1), thus giving an estimated loading concentration of 49.7 ± 23.4 μg/ml or approximately 56%. As the effective synergistic combination of the bactericidal enzymes equates to approximately 8 μg/ml, even at the lower limit of this estimate, this is over 3 times the concentration of the cocktail required to inhibit bacterial growth.

**Fig. 6:**
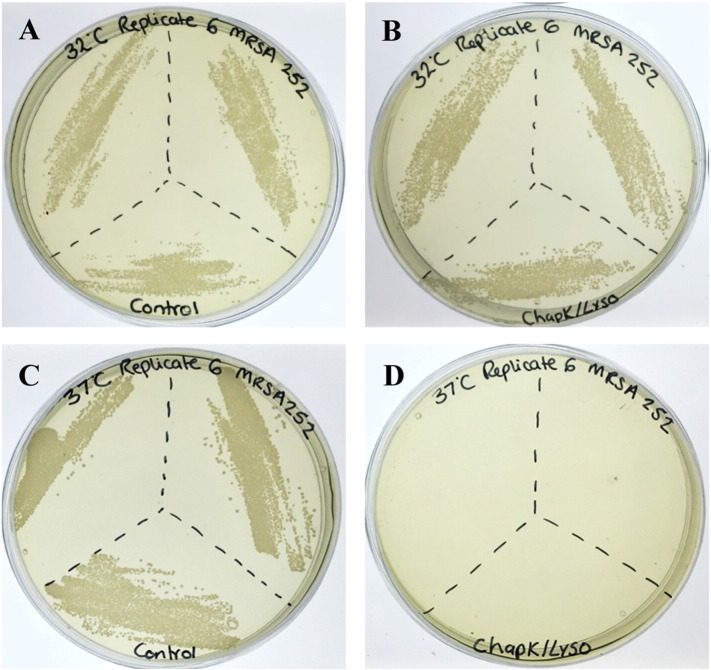
Plate analysis of *S. aureus* MRSA 252 survival and growth - (A) PNIPAM control at 32 °C (B) PNIPAM/CHAP_K_/lysostaphin at 32 °C (C) PNIPAM control at 37 °C (D) PNIPAM/CHAP_K_/lysostaphin at 37 °C.

PNIPAM nanoparticles are able to deliver an enzymatic formulation in a temperature controlled manner as shown by evaluating the difference in cell count post incubation at temperatures both above and below the LCST. Samples plated after incubation at 32 °C demonstrate very little difference in the number of colonies between the controls (nanoparticles without enzybiotic cocktail) (Fig. 6A) and the samples containing the enzybiotic cocktail (Fig. 6B). The control experiments also confirm that the nanoparticles alone do not affect the bacteria. Whereas samples tested at 37 °C indicate a drastic decrease in the number of viable cells post treatment (Fig. 6D). Fig. 6C confirms that the collapse of the nanoparticles above the LCST does not affect the survival of the bacteria, being comparable to the controls undertaken at 32 °C.

**Fig. 7:**
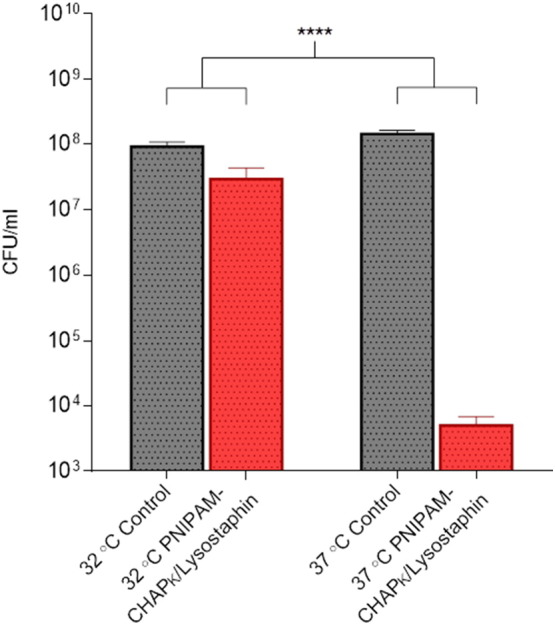
Comparison of bacterial survival at 32 °C and 37 °C for PNIPAM nanoparticle entrapped CHAP_K_/lysostaphin cocktail, relative to PNIPAM nanoparticles without enzymatic cocktail (control) **** *p* < 0.0001.

Quantitative analysis (colony counting) showed a significant difference in the number of viable cells at the two temperatures (*p* ≤ 0.0001, Student's *t*-test). Whilst there is a relatively small change in the number of CFU/ml between the control and the experimental sample at 32 °C, this is not unreasonable when taking into account possible passive diffusion or leaching of the enzymes from the particles. Nonetheless, when compared to samples incubated at 37 °C there is a clear difference in survival, manifesting as a > 4 log reduction in CFU/ml compared to < 1 log difference at 32 °C (Fig. 7).

## 4 Conclusion

In summary this study demonstrates a new and previously unreported application of a novel enzybiotic cocktail for the thermally triggered control of *Staphylococcus aureus*. As potentially viable alternatives to antibiotic therapy, the use of enzybiotics may indicate a possible future treatment for drug resistant bacterial infection. Through exploitation of the ability of lysostaphin to inhibit bacterial growth at very low concentration, and the fast acting nature of the bacteriophage encoded endolysin CHAP_K_, the two antimicrobial enzymes have been shown to work synergistically in the inhibition and subsequent lysis of bacterial cells, demonstrating a faster response time when compared to the current antibiotic of choice for the treatment of MRSA. Moreover, restricting the administration of such antimicrobials by means of an external trigger (in this case an increase in skin temperature associated with infection), limits the possibility of the development of resistance through prevention of any sub-lethal selection pressure. Through exploitation of the thermoresponsive behaviour of PNIPAM, nanoparticles were employed as a drug delivery system capable of releasing their antimicrobial cargo at a biologically relevant temperature indicative of infection. Which, when compared to the same system containing whole phage, the use of an enzybiotic cocktail which is not self-replicating but relies solely on successful diffusion from the polymer matrix and full retention of stability, the results are a promising step forward in the controlled release of an alternative antimicrobial formulation, whilst avoiding any issues associated with the administration of whole phage [32]. Although the clinical indication of our delivery system would be chronic wound infection via utilisation of a dressing/bandage concept, the technology presented here offers the possibility of infection control through use of enzybiotics for a range of conditions owing to the proven stability and retention of activity of the proteins.

## Figures and Tables

**Table 1:** Concentration of protein added to modified fabric, residual non-adsorbed protein and hence encapsulated protein concentration.

Protein added μg/ml	Residual non-absorbed protein μg/ml	Encapsulated protein μg/ml
88.0 ± 9.2	38.3 ± 21.5	49.7 ± 23.4

## Appendix A Supplementary data

Supplementary figures Image 2
