# Supplementary material for: Thermally triggered release of the bacteriophage endolysin CHAPK and the bacteriocin lysostaphin for the control of methicillin resistant Staphylococcus aureus (MRSA)
Source: J Control Release. 2017 Jan 10;245:108–15. doi: 10.1016/j.jconrel.2016.11.030 (PMC5234552; doi:10.1016/j.jconrel.2016.11.030)
Supplement: Supplementary file 1 — Supplementary figures [file mmc1.docx]

Supporting Information

**Thermally Triggered Release of a Synergistic Combination of the Endolysin CHAP_K_ and Lysostaphin for the Control of *Staphylococcus aureus* from Nanoparticle Modified Non-woven Polypropylene**

Hollie Hathaway^1^, Jude Ajuebor^2^, Liam Stephens^1^, Aidan Coffey^2^, Ursula Potter^3^, J. Mark Sutton^4^, A. Toby A. Jenkins^1^*

1. Department of Chemistry, University of Bath, UK, BA2 7AY

2. Department of Biological Sciences, Cork Institute of Technology, Ireland, T12 P928

3. Microscopy and Analysis Suite, University of Bath, UK, BA2 7AY

4. Technology Development Group, Public Health England, Porton Down, UK, SP4 0JG

*Corresponding Author: Email: [a.t.a.jenkins@bath.ac.uk](mailto:a.t.a.jenkins@bath.ac.uk), Tel: +44 (0) 1225 386118

**S1**: FT-IR confirming addition of carbonyl functionality (peak at 1782cm^-1^) to non-woven polypropylene through plasma deposition of maleic anhydride

**S2**: Reduction in turbidity of planktonic MRSA 252 cells (measured as change in OD at 600nm) over a 1 minute period as a function of CHAP_K_ concentration. Tangents fitted in order to calculate initial rate of reaction. Each data point corresponding to the mean of 3 individual experiments and error bars representing the standard deviation.
